# Person-centered hospital care in Indonesia: the role of digital health competency, user interface quality, and user experience in a multi-hospital study

**DOI:** 10.3389/frhs.2026.1827781

**Published:** 2026-06-09

**Authors:** Enny Rachmani, Evina Widiawati, Deshinta Arrova Dewi, Syed Abdul Shabbir, Fu-Yu Chen, Arif Kurniadi, Sylvia Anjani, Eti Rimawati, M. G. Catur Yuantari, Amiq Fahmi

**Affiliations:** 1 Faculty of Health Science, Universitas Dian Nuswantoro, Semarang, Indonesia; 2 Department of Biomedical Engineering, Chung Yuan Christian University, Taoyuan City, Taiwan; 3 Faculty of Data Science and Information Technology, INTI International University, Nilai, Malaysia; 4 Graduate Institute of Biomedical Informatics, College of Medical Science and Technology, Taipei Medical University, Taipei, Taiwan; 5 Faculty of Computer Science, Universitas Dian Nuswantoro, Semarang, Indonesia

**Keywords:** digital health literacy, health system accessible, Indonesia, person-centered, public health, user experience, user interface

## Abstract

**Introduction:**

Hospital digitalization is expected to strengthen person-centered care, yet the everyday experience of healthcare workers depends on both digital readiness and the usability of the systems they operate. This study examined how digital health competency and perceived user interface quality relate to user experience during routine hospital digital work in Indonesia.

**Methods:**

A cross-sectional survey was conducted among healthcare workers from 20 hospitals in Semarang, Indonesia (*n* = 704). Digital health competency was measured using a short public health informatics instrument. User interface quality was measured using a healthcare-oriented interface and usability questionnaire, and user experience was measured using a standardized user experience questionnaire. A prespecified mediation framework tested whether user interface quality mediated the association between digital health competency and user experience. Analyses were complemented with hospital random-intercept multilevel models to account for clustering, and heterogeneity was explored by professional group. Hospital characteristics (public vs. private ownership and related specifications) were examined as organizational factors in adjusted models and descriptive comparisons.

**Results:**

Perceived user interface quality was the strongest and most proximal correlate of user experience. Digital health competency showed no detectable direct association with user experience and no indirect association through user interface quality. Hospital-level clustering explained only a small share of the variance in user experience, indicating that most variability occurred at the individual level. Item-level user interface quality responses highlighted actionable priorities—especially system stability (fewer errors), visibility of system status, and in-context help and error-recovery support.

**Conclusion:**

Improving user interface quality appears to be a higher-leverage pathway for strengthening user experience and enabling person-centered workflows than generic digital upskilling alone. Digitalization strategies should treat usability as core infrastructure work by embedding human-centered requirements into procurement, acceptance testing, and continuous post-implementation improvement tailored to profession-specific workflows and organizational context.

## 1 Introduction

Person-Centered Care (PCC) constitutes a fundamental principle of healthcare quality. The Institute of Medicine's Crossing the Quality Chasm report elucidates patient-centeredness as care that acknowledges and responds to individuals’ unique preferences, requirements, and values, thereby ensuring that patient values inform all clinical decision-making (1). Building upon this foundational premise, the World Health Organization's (WHO) Framework on Integrated People-Centered Health Services (IPCHS) advocates a transition from disease- and institution-centric models to services that are co-produced with people, coordinated across the continuum of care, and designed to promote meaningful engagement of service users (2). As hospitals increasingly digitize clinical and administrative work, PCC is therefore shaped not only by clinical practice but also by how digital tools mediate information access, coordination, and communication at the point of care.

Simultaneously, the rapid advancement of digital technologies has created novel opportunities and concomitant challenges for implementing PCC. The WHO Global Strategy on Digital Health 2020–2025 articulates an aspirational framework for people-centered digital health, urging nations to enhance governance, workforce competencies, interoperability, and evidence generation so that technological innovations substantially improve access, quality, and the patient experience of care (3). In Indonesia, the current wave of hospital digitalization is being accelerated by the national SATUSEHAT initiative, launched in 2022 by the Ministry of Health, which serves as a national platform to integrate and standardize health data exchange across health facilities and digital applications.

SATUSEHAT is positioned as a health information exchange that connects hospitals, primary care facilities, laboratories, pharmacies, and partner applications through nationally defined interoperability specifications, with the explicit goal of enabling more efficient data sharing and continuity of care when patients move between facilities (4, 5). Conceptually, SATUSEHAT aligns with people-centered service goals because integrated, interoperable records can reduce administrative friction, avoid unnecessary repetition of tests, and make care more coordinated around the person, consistent with the WHO's call to reorient services towards integrated, people-centered care and to place people at the center of digital health implementation (2).

However, realizing these person-centered benefits depends not only on infrastructure and standards but also on the day-to-day usability of the systems that healthcare workers must operate, making workforce capability and interface quality central to the success of hospital digital transformation (6). Human factors and socio-technical research shows that poor usability can disrupt workflows, increase cognitive burden, and contribute to error; a recent critical review highlights the need for multi-site evidence linking interface design, user interaction, and system performance in real clinical settings (7). These insights underscore the need to study hospital digitalization not only as a technology deployment but also as a socio-technical intervention embedded in everyday work.

Digital capability frameworks assert that competence extends beyond mere technical abilities to encompass attitudes towards innovation, data utilization, governance, and adherence to safe and ethical practices, all of which are essential prerequisites for person-centered, digitally supported healthcare delivery (8–10). Nevertheless, a study among hospital-based providers has revealed that, while individual eHealth literacy tends to be satisfactory, there is a paucity of evidence in domains requiring interaction with complex systems and organizational access, underscoring enduring frontline obstacles to effective eHealth implementation (11).

Concurrently, User Experience (UX) and the usability of clinical systems, particularly electronic medical records (EMR), are consistently correlated with workload, satisfaction, and the overall well-being of clinicians and nurses. Studies indicate that enhanced EMR usability is associated with reduced task load and diminished likelihood of burnout, with task load serving as a mediating factor in the usability. Thus, suboptimal UX is not merely a technical concern; it can undermine the clinician–patient relationship, the quality of communication, and the experiential dimensions central to PCC (12, 13). Digital services may also support person-centered goals such as continuity and coordination, but they can weaken human connection if design and implementation do not prioritize user-centered values (14). Together, these strands of evidence suggest that digital capability and interface quality should be examined jointly as foundations for person-centered hospital workflows.

Recent studies focused reviews indicate that research on “digital readiness” among hospital staff still overemphasizes individual eHealth or digital health literacy while underestimating domains that depend on interaction with the health information system and on access conditions within facilities (e.g., workflow integration, system access, and practical constraints in daily use). In parallel, a recent critical review of human factors in digital healthcare systems concludes that interaction design and real-world usability are central determinants of system performance, yet many studies remain methodologically limited and insufficiently connected to routine clinical contexts, reinforcing the need for more rigorous, context-sensitive evidence (7, 15).

Despite this, the empirical evidence base remains fragmented. Existing studies in Indonesia and similar settings often focus on digital literacy readiness, acceptance of specific applications, or single-site evaluations, and rarely integrate digital competency, interface quality, and user experience within a single explanatory framework across multiple hospitals (16–19). Moreover, many studies do not explicitly account for the nested structure of hospital data (users clustered within hospitals) or for heterogeneity among professional groups that perform different tasks and face distinct workflow constraints. To date, robust multi-hospital evidence that simultaneously models digital competency, interface quality, and user experience, while considering professional heterogeneity and hospital-level clustering, remains limited in Indonesia. Unlike digital health literacy studies that focus on individuals’ ability to access and understand digital health information, this study operationalizes competency as the capability to perform routine hospital digital tasks within organizational workflows and access constraints, and tests how competency and interface quality jointly shape user experience.

To address these gaps, this multi-hospital study in Indonesia aims to: (i) describe digital health competency, perceived interface quality, and user experience among healthcare workers using routine hospital digital applications; (ii) test a prespecified model in which digital health competency is linked to user experience directly and indirectly through perceived interface quality; and (iii) assess whether these relationships vary by professional group and hospital characteristics. This study contributes (a) theoretically by linking person-centered care and human factors perspectives to routine hospital digital work, (b) methodologically by combining mediation analysis with multi-hospital clustering assessment, and (c) practically by identifying actionable interface priorities that can be embedded into procurement, acceptance testing, and continuous improvement within real-world healthcare infrastructure projects.

## 2 Materials and methods

**Figure 1:**
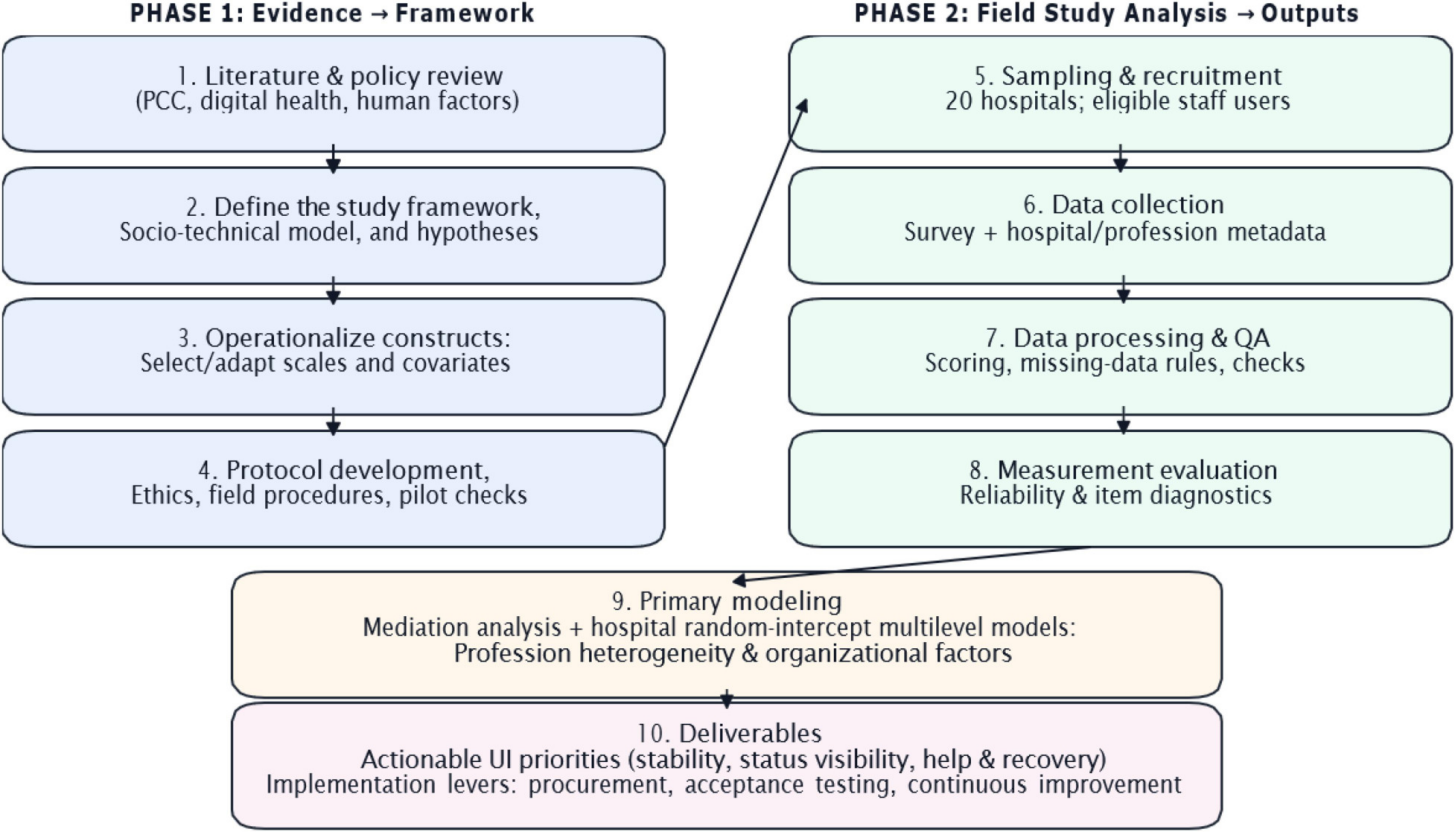
Methodological flow diagram.

This section describes the study design, implementation procedures, and analytic strategy in a structured and reproducible manner. Because the work combines framework development (grounded in person-centered care and human factors literature) with a multi-hospital field survey and hierarchical statistical modeling, Figure 1 provides an overview of the end-to-end workflow before detailing each component. The figure summarizes the methodological flow from evidence synthesis and framework specification, through instrument operationalization and protocol development, to multi-site sampling, data collection, measurement evaluation, and the prespecified modeling strategy (mediation analysis complemented by hospital-level random-intercept models).

### 2.1 Study design and reporting

We conducted a multi-site, cross-sectional survey of healthcare workers across 20 hospitals in Semarang, Indonesia. The survey was conducted from February to November 2023. The reporting of this study follows the STROBE (STrengthening the Reporting of OBservational studies in Epidemiology) Statement for observational studies (20).

### 2.2 Setting and participants

Hospitals were selected purposively based on willingness to participate and to reflect variability in ownership, teaching status, and digitalization maturity. Eligible participants were healthcare professionals (e.g., physicians, nurses, medical record officers) who used hospital digital applications (e.g., EMR/Hospital Information System) in their routine work. Data were collected from 704 respondents who met the inclusion criteria of being≥18 years old, employed at a participating hospital, and self-reporting use of relevant hospital digital systems in the last 3 months; exclusion criteria included interns/trainees without system access and respondents who had not completed the questionnaire.

### 2.3 Ethics and consent

The protocol received ethics approval No. 328/EA/KEPK-Fkes-UDINUS/XI/2022. Participation was voluntary, and respondents provided informed consent prior to survey administration. Data were de-identified prior to analysis.

### 2.4 Measures

This study employs a psychometric evaluation using valid and reliable Likert-scale questionnaires (21). The questionnaire comprises the User Experience Questionnaire (UEQ) (22, 23) to measure user experience and the Public Health Informatics Competencies for Primary Healthcare Short Version (PHIC4PHC-S) Questionnaire to assess digital health competencies (10, 24). and the User Interface Quality Healthcare System Questionnaire (UIQHS) to evaluate user interface quality (25, 26). All instruments have demonstrated validity in prior studies. In the present sample, we additionally evaluated internal consistency (Cronbach's *α*) with 95% Confidence Intervals (CI) for each scale/subscale to support the reliability of the scores used in subsequent analyses. Item–rest correlations and *α* if the item was deleted were examined to identify items with weak discrimination.

In this study, for subgroup summaries and regression analyses, summed scale scores were used. UX was computed as the sum of 26 UEQ items scored 1–7 after reverse-coding negatively oriented items (theoretical range 26–182). User Interface quality (UI) was computed as the sum of 22 UIQHS items, each scored 1–5 (range: 22–110). Digital Health Competency (DHC) was computed as the sum of 12 PHIC4PHC-S items scored 1–5 (range 12–60). UEQ raw scores were additionally transformed to the conventional −3 to +3 metric as a linear rescaling for visualization only.

#### 2.4.1 Digital health competency

This study measured DHC using PHIC4PHC-S, derived from the validated 42-item PHIC4PHC instrument. The original and short versions include domains of cognitive, technical, and ethical proficiency, as well as health information literacy. Higher scores indicate greater competency (10, 24). Internal consistency of the DHC was assessed using Cronbach's alpha (*α*) with 95% confidence intervals and item diagnostics. Construct validity was explored using confirmatory factor analysis (CFA; Maximum Likelihood estimator), with model fit evaluated using the Comparative Fit Index (CFI), Tucker–Lewis Index (TLI), Root Mean Square Error of Approximation (RMSEA-90% CI), and Standardized Root Mean Square Residual (SRMR). Because the short form retained an uneven number of items across domains (including a single-indicator domain), a fully identified four-factor CFA was not feasible. Therefore, this study applied a theory-driven domain-merging approach to obtain an identified three-domain CFA model for construct checking. Negatively worded items were reverse-coded prior to analysis. For hypothesis testing in mediation and mixed-effects models, the analysis used the DHC composite score to preserve comparability with prior scoring and to maintain the prespecified definition of the predictor.

#### 2.4.2 User experience

UX with hospital digital systems was assessed using the UEQ, which covers Attractiveness through Innovative. UEQ Questionnaire, administered on a 7-point semantic differential format. Responses were recorded as 1–7 in the raw dataset; items with negative polarity were reverse-coded so that higher values consistently indicated more positive UX. In addition, the same raw item responses were processed using the official UEQ analysis tool, which transforms scores to the standard range of −3 to +3. The −3 to +3 values are therefore a standardized representation of the raw responses for visualization and interpretation with the UEQ benchmark interpretation, not a separate measurement scale (22). Furthermore, for regression/mediation and mixed-effects models, this study used UEQ scale scores after reverse coding. Internal consistency of the UX measure in the present sample was evaluated using Cronbach's alpha (*α*) with 95% confidence intervals and item diagnostics (item–rest correlations; *α*-if-item-deleted). Because UEQ is an established instrument, and the primary aim of this study is predictive modeling (DHC → UI → UX), this study reports sample-based reliability evidence and uses the prespecified UEQ composite scores in the mediation and mixed-effects analyses.

#### 2.4.3 User interface quality

To evaluate interface-specific qualities complementary to UIQ, this study administered a UIQHS with 5-point Likert responses from “strongly disagree” to “strongly agree” (26). UIQHS indicators were grounded in Nielsen's 10 usability heuristics with context relevant extensions and included: Visibility of system status, Match between system and real world, User control and freedom, Consistency and standards, Error prevention, Flexibility and efficiency of use, Help users recognize, diagnose, and recover from errors, plus Eye catching (aesthetic appeal), Privacy & security, and Help/Documentation (27, 28). The construct validity of the UIQHS was examined using confirmatory factor analysis (CFA) with maximum likelihood estimation. Model fit was evaluated using *χ*^2^, CFI, TLI, RMSEA (90% CI), and SRMR. Internal consistency reliability was assessed using Cronbach's alpha (*α*) with 95% confidence intervals and item diagnostics (item–rest correlations; *α* if item deleted).

#### 2.4.4 Covariates

Pre-specified covariates included professional group (physicians, nurses, medical record officers), gender, education level, computer training, motivation using internet, and hospital type (public/private).

### 2.5 Questionnaire administration

Data were collected on paper at participating wards/units over 12 months. Instruments were in Bahasa Indonesia; localized items underwent translation and back-translation, as well as cognitive debriefing, to ensure semantic and conceptual equivalence prior to the main data collection (20).

### 2.6 Data management and quality assurance

Paper forms were entered into the JASP statistical program and subjected to logic checks. *A priori* rules addressed straight-lining and implausible response patterns; cases with excessive missingness on primary scales were excluded. For item-level missingness ≤10%, we used person-mean imputation within scales; sensitivity analyses compared complete-case and imputed results (20).

### 2.7 Statistical analysis

All analyses were prespecified to evaluate the integration of DHC, UI, and UX models and are reported in accordance with STROBE recommendations for observational studies (20, 29). This study used the JASP statistical application (RRID:SCR_015823) for statistical analysis. This manuscript first reported descriptive statistics for participant and hospital characteristics, as well as scale score distributions.

Mediation analysis was estimated using a Structural Equation Modeling (SEM) framework implemented in JASP (lavaan) with maximum likelihood (ML) estimation. This study specified DHC as the predictor, UI as the mediator, and UX as the outcome, while adjusting for prespecified covariates (hospital type, professional group, education, computer training, and internet use). Indirect effects were tested using bias-corrected bootstrapping with 5,000 resamples, and results are reported as standardized coefficients (*β*) with 95% confidence intervals (30).

To account for the nested structure of the data (respondents clustered within hospitals), this study conducted complementary hospital random-intercept mixed-effects models using Hosp_ID as the random-effects grouping factor. The first step involved fitting intercept-only (null) models for UX, UI, and DHC to estimate variance components and Intraclass Correlation Coefficients (ICCs), then fitting an adjusted hospital random-intercept model for UX, including UI, DHC, and the prespecified covariates, to quantify residual between-hospital variance after adjustment. These multilevel models were used to confirm that the key associations (UI→UX and DHC→UX) were robust to hospital-level clustering. Because the SEM mediation was estimated at the individual level, this study interprets indirect-effect inferences with appropriate caution and reports the multilevel results (variance components/ICC) as complementary evidence that addresses clustering.

Assumption checks for this study are as follows: the primary regression specifications (UX and UI equations). This study assessed multicollinearity using tolerance and Variance Inflation Factors (VIF). Residual diagnostics were inspected using standardized residual histograms and Q–Q plots to evaluate residual normality, and residuals-vs.-predicted plots to evaluate linearity and homoscedasticity. Potential extreme outliers were screened using standardized residual ranges.

## 3 Results

### 3.1 Respondent characteristics

A total of 704 healthcare workers from 20 hospitals were analyzed (Table 1). Most respondents worked in public hospitals (57.4%), and female staff predominated (73.3%). The largest education stratum was Diploma holders (55.4%), followed by Bachelor's degree holders (34.7%), while Master's degree holders were few (1.3%). By profession, nurses comprised the majority (57.2%), followed by medical record officers (40.9%); physicians represented 1.8% (*n* = 13). Two-thirds (65.1%) reported non-formal computer training. Most internet use is for entertainment, communication, and work.

**Table 1:** Respondents characteristic.

Category	*N*	%	User interface evaluation			Digital health competence			User experience		
			M	SD	*p*	M	SD	*p*	M	SD	*p*
Hospital Specification											
Public	404	57.4	86.9	9.8	.270	49.4	5.6	.863	129.5	22.6	.262
Private	300	42.6	86.3	10.5		49.1	6.1		127.8	21.2	
Gender											
Male	188	26.7	86.9	9.8	.292	50.2	4.9	.011	127.8	21.8	.553
Female	516	73.3	86.5	10.3		48.8	6.2		129.1	22.1	
Education											
High School Education	61	8.7	86.8	9.4	.819	49.9	4.7	.701	127.8	21.3	.346
Diploma Education	390	55.4	86.6	9.8		48.9	5.6		128.4	22.1	
Bachelor Education	244	34.7	86.3	10.6		49.4	6.4		128.7	21.8	
Master Education	9	1.3	94.8	12.5		51.2	7.9		148.9	20.3	
Profession											
Medical Record Officers	288	40.9	86.6	9.8	.026	51.4	4.5	<.001	128.3	21.5	.313
Nurses	403	57.2	86.4	10.3		47.7	6.2		128.7	22.3	
Physician	13	1.8	94.1	10.5		50.2	6.8		137.8	23.1	
Computer Training											
No	458	65.1	87.1	10.1	.220	49.1	5.9	.520	128.8	22.1	.792
Yes	246	34.9	85.8	10.2		49.6	5.6		128.6	21.8	
Motivation using the internet											
Education (No)	342	48.6	86.7	9.9		49.4	6.1		131.3	22.8	
Education (Yes)	362	51.4	86.6	10.4		49.0	5.5		126.3	21.0	
Shopping (No)	273	38.8	86.1	10.6		49.3	6.0		128.1	22.7	
Shopping (Yes)	431	61.2	87.0	9.8		49.2	5.7		129.2	21.6	
Entertaining (No)	187	26.6	85.4	10.3		49.2	6.4		128.1	23.9	
Entertaining (Yes)	517	73.4	87.1	10.0		49.2	5.6		129.0	21.3	
Working (No)	267	37.9	85.7	9.8		49.5	5.1		126.6	20.8	
Working (Yes)	437	62.1	87.2	10.3		49.0	6.2		130.0	22.6	
Communication (No)	178	25.3	84.6	10.5		49.0	6.0		126.3	22.2	
Communication (Yes)	526	74.7	87.3	9.9		49.3	5.8		129.6	21.9	
Seeking general information (No)	311	44.2	85.9	10.1		49.5	5.6		126.3	20.9	
Seeking general information (Yes)	393	55.8	87.2	10.1		49.0	6		130.7	22.6	
Seeking Health Information (No)	415	58.9	86.1	10.4		49.4	5.7		127.7	22.3	
Seeking Health Information (Yes)	289	41.1	87.4	9.7		48.9	6.0		130.2	21.5	
Wasting time (social media) (No)	341	48.4	86.0	10.5		49.0	6.2		127.9	22.6	
Wasting time (social media) (Yes)	363	51.6	87.2	9.7		49.4	5.4		129.5	21.4	

*M* = mean; SD = standard deviation. Two-level comparisons used independent-samples t tests; comparisons across ≥3 groups used one-way ANOVA, with Welch's ANOVA reported when homogeneity of variance was violated. *p*-values are shown without leading zeros; exact *p*-values are reported unless *p* < .001. Internet-use motivations are descriptive only (no hypothesis tests).

Regarding internet use motivations, work-related use was associated with the highest UX (130.1) and slightly higher UI (87.2), whereas education-related use showed the lowest UX (126.3) and comparable UI (86.6). Taken together, the sample is dominated by public-sector, nursing, and diploma-educated staff, with largely consistent UI and DHC across strata and modest UX differences by hospital ownership, sex, and reported internet-use motivations.

Group differences in digital health competency, user experience, and user interface quality were examined using independent-samples t tests for two-level factors (computer training, sex, and hospital ownership/specification) and one-way ANOVA for profession (three groups). For computer training, there were no significant differences in digital health competency, t(702) = −1.16, *p* = .246, user experience, t(702) = 0.10, *p* = .923, or user interface quality, t(702) = 1.49, *p* = .137. Sex was associated with digital health competency, t(702) = 2.70, *p* = .007, but not with user experience, t(702) = −0.67, *p* = .500, or user interface quality, t(702) = 0.50, *p* = .618. Hospital ownership/specification was not associated with digital health competency, t(702) = 0.63, *p* = .532, user experience, t(702) = 1.00, *p* = .318, or user interface quality, t(702) = 0.73, *p* = .464.

Profession-level differences were tested with ANOVA. For user experience, the omnibus test was not significant, F (2, 701) = 1.16, *p* = .313 [Levene's F (2, 701) = 0.28, *p* = .757]. For user interface quality, the profession effect was significant, F(2, 701) = 3.68, *p* = .026 [Levene's F (2, 701) = 1.52, *p* = .219]. For digital health competency, the homogeneity assumption was violated [Levene's F (2, 701) = 3.63, *p* = .027]; therefore, Welch's ANOVA was used and indicated a significant profession effect, Welch's F (2, 32.14) = 39.64, *p* < .001. Descriptive means by group are presented in Table 1.

### 3.2 User interface quality evaluation

This study employed the UIQHS questionnaire to assess UI. The questionnaire consists of 22 questions covering 10 usability heuristics. UIQHS showed excellent internal consistency (Cronbach's *α* = 0.929, 95% CI 0.920–0.939). CFA supported the proposed multi-factor structure with acceptable model fit [CFI = 0.917, TLI = 0.903, RMSEA = 0.072 (90% CI 0.067–0.078), SRMR = 0.049]. Standardized factor loadings were significant for all indicators (*p* ≤ .001), with most loadings in the moderate-to-high range (approximately 0.47–0.86), supporting convergent validity for the majority of items.

Overall, UI evaluation remained consistent across subgroups (mean 86–87). Notable deviations were observed among Master's degree holders (94.8) and physicians (94.1), although both groups had very small sample sizes (Table 1).

Table 2 shows that across the 22 statements (5-point scale), respondents reported consistently positive perceptions of the interface, with item means clustering around 4.0 (range 3.10–4.29). The highest ratings centered on clarity and language: “*each user has a username and a password”* (mean 4.29), “*the system uses Indonesian, which is easy to understand”* (4.16), and “*everything the system displays can be understood clearly”* (4.10). Logical organization and consistency were also strong (e.g., 4.05 for both *logical data sequence* and *consistent naming of controls/icons*), indicating that navigation, terminology, and information flow are generally well aligned with user expectations.

**Table 2:** Mean of responses of user interface quality evaluation.

No	Statements	($\bar{x}$)	Std. Deviation
1	Everything the system displays can be understood clearly	4.10	0.590
2	The system always displays indicators in the form of progress bars or other things, if there are changes	3.69	0.724
3	The system is designed so that users can easily identify the function of each available control/icon	4.00	0.668
4	The most frequently used functions are found at the top/main system	4.02	0.651
5	The system uses Indonesian, which is easy to understand	4.16	0.690
6	The sequence of data in the system has been arranged logically	4.05	0.645
7	Similar items/data become one	4.02	0.671
8	The system provides a button to return to the main menu on each input screen	4.02	0.708
9	The system provides buttons to undo and redo actions that have been performed	4.01	0.721
10	The system provides a warning when leaving the input page	3.91	0.791
11	All saved actions/input can be accessed at any time	4.02	0.685
12	The naming of control buttons/icons on the system is made consistently	4.05	0.633
13	The system is designed to be familiar because it is made in accordance with general system standards	3.95	0.719
14	The system never experiences problems or errors during use	3.10	1.097
15	The system display, both in the form of images and links, functions properly	3.94	0.695
16	The system is able to display or hide the required information	4.02	0.661
17	The system always notifies the location of the error and provides appropriate input suggestions if the user makes an input error	3.75	0.843
18	The system provides a help button when the user does not know how to input data	3.61	0.924
19	The system has the ability to expand the work area	3.96	0.664
20	Each user has a username and a password	4.29	0.696
21	All required documents can be accessed	3.99	0.726
22	Area fills in questions as needed	4.00	0.650

**Figure 2:**
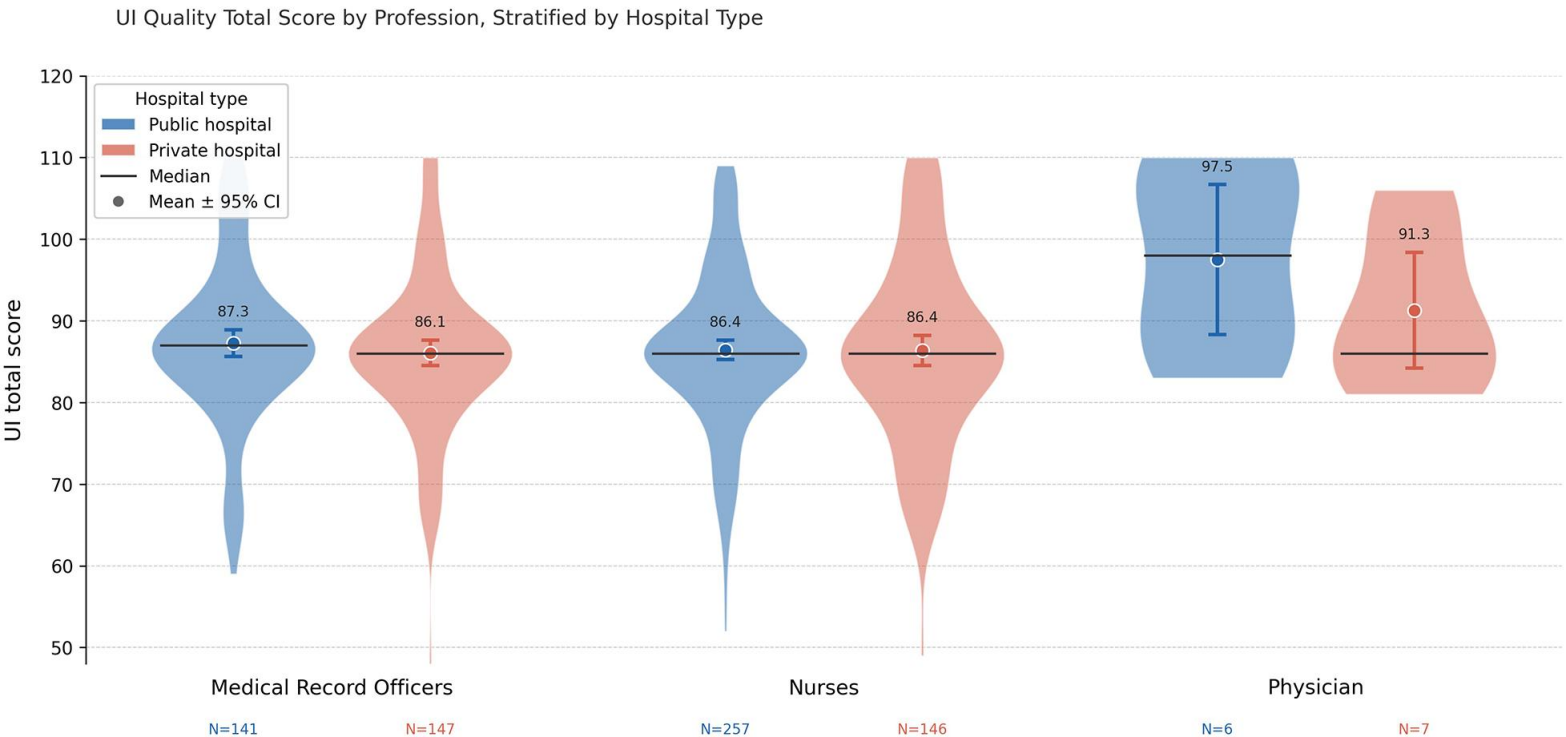
Score of user interface quality evaluation among professionals and hospital types.

In contrast, areas for improvement involved system robustness and in-flow assistance. The lowest score was for *freedom from problems or errors during use* (3.10), while *help availability when users do not know how to input data* (3.61), *visibility of status/progress indicators* (3.69), and *error localization with corrective suggestions* (3.75) were modest and dispersion was also greatest on these items (e.g., SD 1.10 for stability; SD 0.92 for help),

Figure 2 shows UI total score distributions by profession stratified by hospital type. UI scores are broadly similar between public and private hospitals among medical record officers and nurses, whereas physicians show higher UI scores, though this subgroup is small and should be interpreted with caution. Furthermore, this result is consistent with Table 1 and the one-way ANOVA results, UI differed by profession (*p*=0.026), with physicians tending to report higher UI scores than medical record officers and nurses

### 3.3 Digital health competency

The DHC scale was measured using the PHIC4PHC-S and demonstrated good internal consistency (Cronbach's *α* = 0.843, 95% CI 0.812–0.875). CFA results suggested that DHC is multidimensional rather than strictly unidimensional; however, the global fit of the tested CFA model remained suboptimal [CFI = 0.826, TLI = 0.774, RMSEA = 0.137 (90% CI 0.128–0.146), SRMR = 0.087]. Most indicators showed significant standardized loadings, particularly for questions 6–12 (approximately 0.74–0.84), whereas questions 2 and 3 displayed weak loadings (∼0.25) and low explained variance (R^2^ ∼0.06), consistent with item-level reliability diagnostics. Given the study's predictive focus, we retained the DHC composite score for hypothesis testing and report these measurement findings transparently as a limitation and a target for future scale refinement. The DHC questionnaire that has been used is a short version; it showed good internal consistency but CFA indicated suboptimal global fit, suggesting that a single measurement model may not fully capture the construct in this context

DHC averages clustered around 49–50 in most subgroups, with comparatively higher values in medical record officers (51.4) and the Master's group (51.2) (Table 1).

Overall, Table 3 reported, respondents reported favorable competencies across most items (means clustered around 4.0 on a 5-point scale). The highest agreement centered on information governance and health-information seeking: “I know the importance of confidentiality when processing data” (mean 4.47; SD 0.651), “the internet can be used as a health information resource” (4.35; 0.667), and “I can receive and send emails to transfer files” (4.34; 0.694). Foundational skills were also strong-file management/saving (4.22; 0.686), awareness of regulations on patient identity protection (4.22; 0.750), using computers as a tool for work (4.18; 0.630), and evaluating health information online (4.12; 0.702). Routine use of health information systems was rated positively (3.99; 0.751).

**Table 3:** Mean of responses to digital health competency.

No	Statement	($\bar{x}$)	Std. Deviation
1	I can receive and send emails to transfer files through the network	4.34	0.694
2	I do not know how to use the computer for personal use	3.77	1.198
3	I cannot use a spreadsheet program (e.g., Excel) to perform simple data analysis.	3.54	1.112
4	I can use database software to create a database that is needed for my daily tasks.	3.82	0.905
5	I can use health information systems to complete my work	3.99	0.751
6	I know how to manage and save files	4.22	0.686
7	Computers can be used as a tool for working and controlling	4.18	0.630
8	I know the importance of confidentiality when processing data in the medical records and on computers	4.47	0.651
9	I know the regulations concerning the protection of patient identity on computers	4.22	0.750
10	I know that the internet can be used as a health information resource	4.35	0.667
11	I know how to use the internet to answer questions about health	4.21	0.668
12	I can evaluate health information found on the internet	4.12	0.702

The lowest scores are for spreadsheet use for simple analysis (3.54; SD 1.112), creating databases for daily tasks (3.82; 0.905), and general/personal computer use (3.77; 1.198). Notably, these lower-scoring items also showed the greatest dispersion (largest SDs), indicating heterogeneous proficiency and suggesting that focused, hands-on training (particularly in spreadsheets and basic database functions) could yield the most improvement.

**Figure 3:**
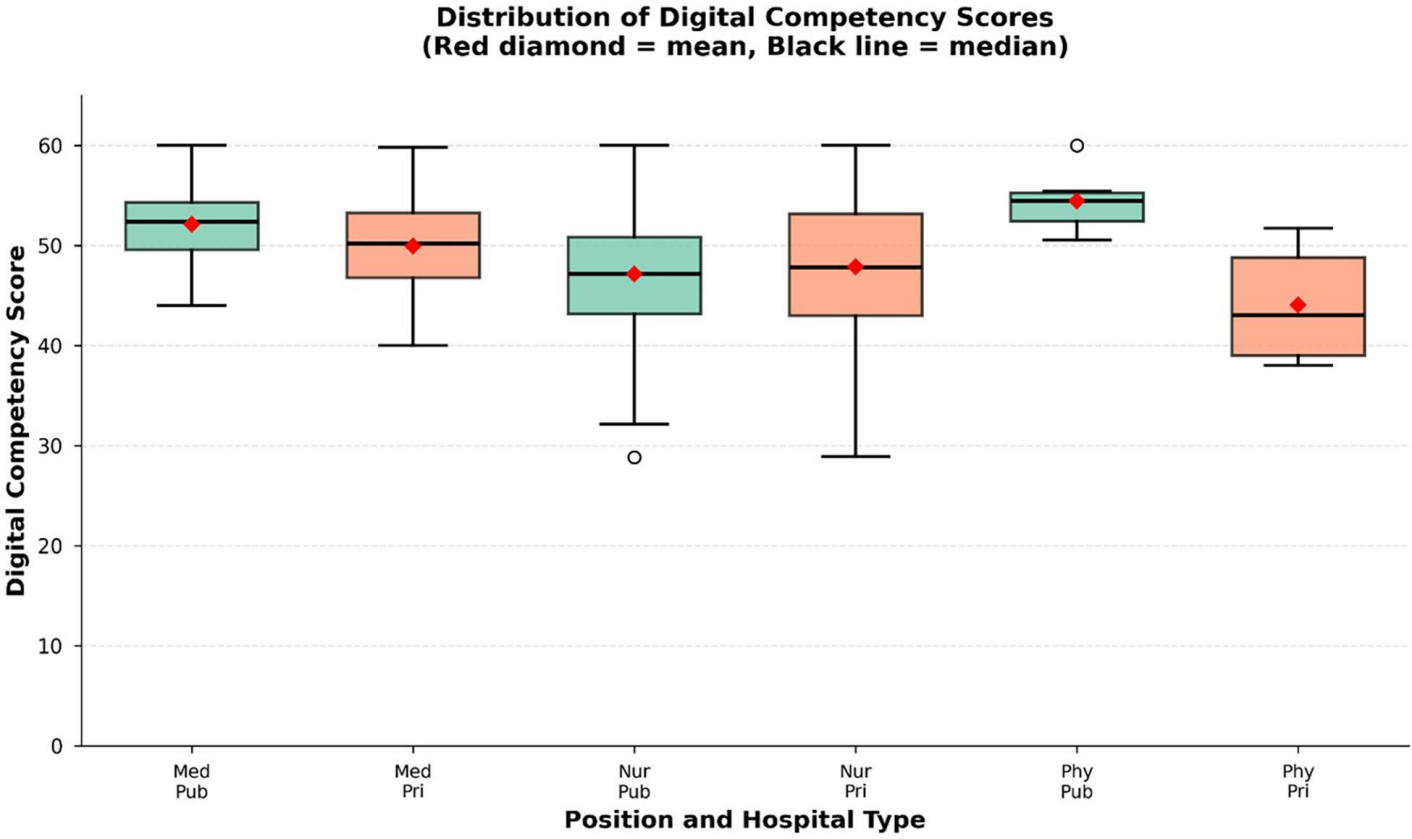
Boxplot distribution of digital health competencies score.

Forest plot (Figure 3) displaying mean digital competency scores (circles) with 95% confidence intervals (horizontal lines) for each position-hospital type combination. Public hospitals are shown in green, private hospitals in orange. The vertical dashed line at 50 serves as a reference point. Sample sizes (*N*) are displayed in the right column. Overlapping confidence intervals suggest no statistically significant differences in most positions between hospital types, though Medical Record Officers in public hospitals show an observable advantage.

### 3.4 User experience

The UEQ was used to measure UX and demonstrated excellent internal consistency in this sample (Cronbach's *α* = 0.954, 95% CI 0.950–0.958). Item-rest correlations were generally moderate to high, supporting coherence of the composite UX score used in subsequent analyses. One item (UX1) showed weaker discrimination (item-rest *r* = 0.171), and *α* increased slightly if this item was removed (*α* = 0.956); we retained all items for primary analyses to preserve content coverage and interpret this item cautiously

Table 1 reveals that the mean UX scores were marginally higher in public than in private hospitals (129.5 vs. 127.8). UX was higher among females (129.1) than among males (127.8), despite similar UI scores between the sexes. Across education, the Master's stratum showed the highest UX (148.9; *n* = 9), while the larger Diploma and Bachelor groups clustered around 128–129. By profession, physicians exhibited higher UX (137.8; *n* = 13) than nurses (128.7) and medical record officers (128.3). UX did not materially differ by computer training (128.8 without vs. 128.6 with training).

**Figure 4:**
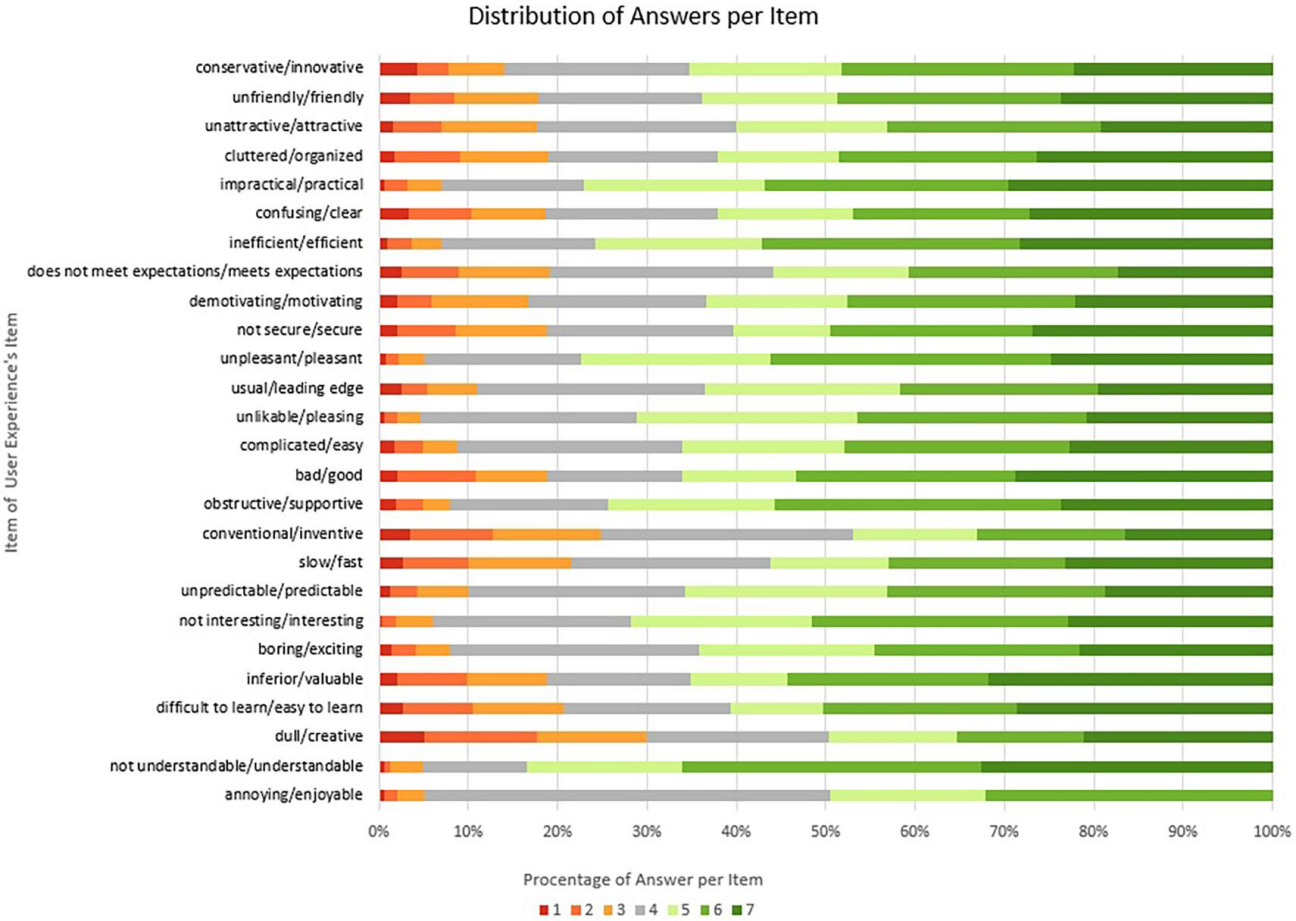
Distribution response per item of the user experience questionnaire.

Figure 4 shows the stacked distribution of UEQ responses for the 26 UEQ items. Across the scale, ratings are strongly skewed to the positive end (categories 5–7, green), with only small negative tails (categories 1–3). Pragmatic qualities score particularly well: users frequently judged the system clear and organized (“confusing/clear”, “cluttered/organized”), efficient (“inefficient/efficient”), and easy to learn/understand (“difficult to learn/easy to learn”, “not understandable/ understandable”). Core valence items such as good, pleasing/likable, supportive, valuable, and enjoyable also concentrate in the highest categories, indicating an overall favorable experience.

**Figure 5:**
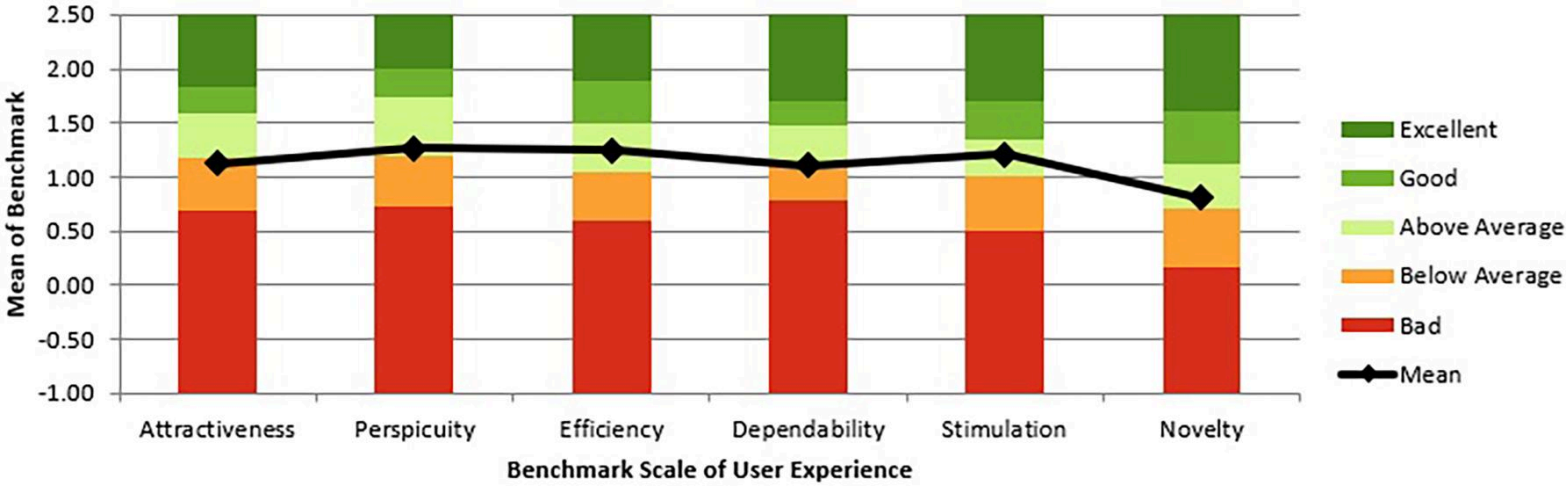
The indicators of user experiences.

Figure 5 benchmarks the six UEQ dimensions on the −3 to +3 scale and shows that all means sit clearly on the positive side (black line range 1.0–1.4). The distribution bars are dominated by “Above average–Excellent” responses (light/dark green), indicating a broadly favorable experience. Among the dimensions, Stimulation attains the highest mean (range 1.3–1.4), followed by Perspicuity and Dependability (range 1.2–1.3), with Efficiency and Attractiveness also comfortably positive (range 1.1–1.2). Furthermore, novelty is the lowest but remains above zero (<1.0).

### 3.5 Statistical analysis

Prior to interpreting regression-based results, this study inspected collinearity and residual diagnostics for the main UX regression specification. Collinearity was negligible (VIF ≈ 1.0 for continuous predictors). Standardized residual diagnostics indicated no major deviation from normality, and residuals-vs.-predicted plots showed no strong patterns indicative of heteroscedasticity/nonlinearity. Standardized residuals ranged from −2.456 to 2.395.

#### 3.5.1 Mediation analysis

The mediation analysis examined whether UI Quality mediates the relationship between DHC and UX. All estimates were reported as standardized coefficients (*β*) with 95% using 5,000 bootstrap resamples (Table 4).

**Table 4:** Mediation analysis among DHC, UI, and UX.

Parameter	Std. estimate	Std. Error	*p*	z-value	95% Confidence Interval	
					Lower	Upper
*Direct effects*						
DHC → UX	−0.044	0.037	−1.195	0.232	−0.117	0.028
*Indirect effects*						
DHC → UI → UX	0.002	0.017	0.091	0.928	−0.030	0.035
Total Effects						
DHC → UX	−0.043	0.041	−1.048	0.295	−0.122	0.038
*Path coefficients*						
UI → UX	0.374	0.035	10.733	<.001	0.306	0.440
DHC → UX	−0.044	0.037	−1.195	0.232	−0.117	0.028
DHC → UI	0.004	0.044	0.091	0.928	−0.081	0.092
Hosp.Spes → DHC	−0.049	0.037	−1.337	0.181	−0.123	0.022
Profession → DHC	−0.340	0.035	−9.729	<.001	−0.410	−0.272
Education → DHC	0.137	0.041	3.362	<.001	0.057	0.220
Training → DHC	−0.019	0.036	−0.542	0.588	−0.092	0.048
Using Internet → DHC	−0.016	0.037	−0.419	0.675	−0.088	0.059
Hosp.Spes → UI	−0.023	0.038	−0.598	0.550	−0.098	0.055
Profession → UI	0.012	0.043	0.285	0.775	−0.072	0.098
Education → UI	9.636 × 10^−4^	0.040	0.024	0.981	−0.077	0.080
Training → UI	−0.046	0.037	−1.230	0.219	−0.117	0.027
Using Internet → UI	0.088	0.038	2.308	0.021	0.011	0.161
Hosp.Spes → UX	−0.027	0.035	−0.755	0.450	−0.094	0.046
Profession → UX	−0.006	0.042	−0.155	0.876	−0.085	0.077
Education → UX	0.039	0.039	1.002	0.316	−0.036	0.115
Training → UX	0.023	0.036	0.638	0.523	−0.045	0.094
Using Internet → UX	0.017	0.034	0.508	0.611	−0.049	0.084

In the prespecified Mediation and path analysis (DHC → UI → UX) model with DHC as the predictor, UI as the mediator, and UX as the outcome, the direct effect of DHC on UX was small and non-significant (standardized *β* = −0.044, SE = 0.037, z = −1.195, *p* = 0.232; 95% CI −0.117, 0.028). The indirect effect via UI was essentially null (*β* = 0.002, SE = 0.017, z = 0.091, *p* = 0.928; 95% CI −0.030, 0.035), yielding a non-significant total effect of DHC on UX (*β* = −0.043, SE = 0.041, z = −1.048, *p* = 0.295; 95% CI −0.122, 0.038). All estimates were obtained using maximum likelihood, and confidence intervals did not include zero, indicating no statistical evidence for the hypothesized mediation in this specification.

**Figure 6:**
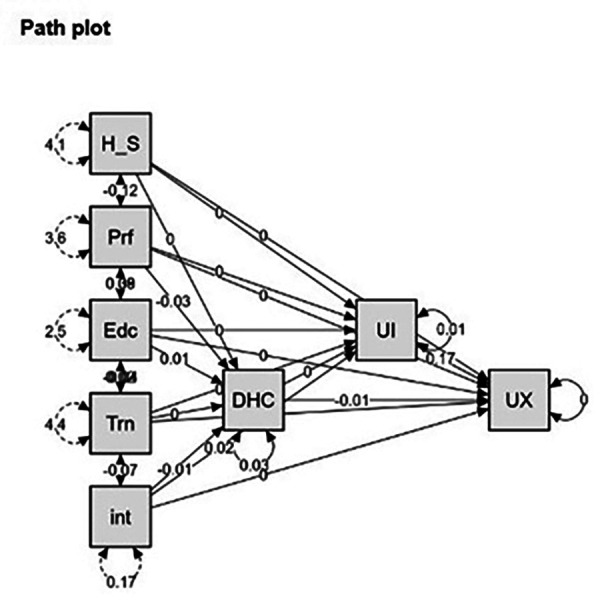
Path coefficient among parameters.

Complementary path coefficients confirmed that perceived UI quality is a strong proximal driver of UX (UI → UX: *β* = 0.374, SE = 0.035, z = 10.733, *p* < .001; 95% CI 0.306, 0.440), while DHC showed no detectable association with either UI (*β* = 0.004, *p* = 0.928) or UX (*β* = −0.044, *p* = 0.232). Among covariates, professional group was strongly associated with DHC (*β* = −0.340, SE = 0.035, *p* < .001; 95% CI −0.410, −0.272), and internet use showed a small positive association with UI (*β* = 0.088, SE = 0.038, *p* = 0.021; 95% CI 0.013, 0.162), whereas hospital type, education, and computer training were not significant (all *p* > .05). Taken together, the model indicates that in this dataset UX varies primarily with perceived interface quality rather than with digital competency, and that DHC does not exert an indirect effect on UX through UI (Figure 6).

#### 3.5.2 Multilevel analysis

This study addresses the dataset’s hierarchical structure (Level 1: individual respondents; Level 2: hospitals) and then quantifies hospital-level clustering and estimates a hospital random-intercept Bayesian linear mixed-effects model (BLMM). We first fitted intercept-only (null) random-intercept models for UX, UI, and DHC to obtain variance components and ICC, defined for a random-intercept model as the ratio of between-hospital variance to total variance (between-hospital + residual variance). Table 5 reports these ICC estimates and the associated variance components.

**Table 5:** ICC from hospital random-intercept models (null and adjusted).

Outcome	Model specification	Var(Hosp_ID intercept)	Var(Residual)	ICC
UX	Null model: UX∼1 + (1|Hosp_ID)	8.544	479.1	0.0175
UI	Null model: UI∼1 + (1|Hosp_ID)	0.953	102.9	0.0092
DHC	Null model: DHC∼1 + (1|Hosp_ID)	4.491	31.41	0.1251
UX	Adjusted model: UX∼UI + DHC + profession + training + education + internet use + hospital type + (1|Hosp_ID)	6.303	415.3	0.0150

Table 5 shows that the null-model ICC for UX was 0.0175, indicating that approximately 1.75% of the variance in UX was attributable to between-hospital differences, with the remainder attributable to within-hospital individual-level variation. DHC showed higher hospital-level clustering (ICC = 0.1251) than UI (ICC = 0.0092). In the adjusted hospital random-intercept model for UX, the ICC remained small (ICC = 0.0150), indicating that most variability in UX occurs at the individual level while still justifying adjustment for hospital clustering

This study used a BLMM to examine the effects of UI and DHC on the dependent variable (UX), with hospital as a random effect to account for the hierarchical structure of the data. Fixed effects included DHC, UI, and the prespecified covariates (professional group, training, education, internet use, and hospital type). Furthermore, to ensure model stability, the Hamiltonian Monte Carlo (HMC) algorithm was used in this study, with multiple chains and sufficient iterations, and 95% credible intervals were reported (8,000 iterations with an adaptive delta of 0.99 and a maximum tree depth of 20). Convergence diagnostics were satisfactory (R-hat ≈ 1.00; effective sample sizes adequate).

After incorporating hospital-level clustering into the analysis, the quality of UI emerged as a significant positive predictor of UX (estimate = 0.807, 95% credible interval 0.656 to 0.958), while DHC exhibited no credible correlation with UX (estimate = −0.141, 95% credible interval −0.416 to 0.134) (Table 6). The covariates examined, including profession, training, education, internet usage, and hospital type, had confidence intervals that included zero, indicating weak or inconsistent evidence of direct relationships with UX in this model specification. The variance components derived from the hospital random-intercept Bayesian linear mixed model (BLMM) revealed a moderate degree of variability among hospitals (Hosp_ID) intercept variance = 6.254; residual variance = 415.2), which aligns with the minimal adjusted ICC reported in Table 5. In summary, the multilevel analysis substantiates the principal conclusion that perceived UI quality serves as the foremost proximal determinant of UX and that DHC does not significantly predict UX, even when accounting for the nested multi-hospital framework (Tables 5, 6).

**Table 6:** Bayesian linear mixed-effects model by hospital.

Parameter	Estimate	Std. Error	95% Confidence Interval		R-hat	ESS (bulk)	ESS (tail)
			Lower	Upper			
Intercept	62.71	11.31	40.21	85.35	1.000	27,160	13,620
DHC (differences from intercept)	−0.141	0.141	−0.416	0.134	1.000	25,190	13,330
UI (differences from intercept)	0.807	0.078	0.656	0.958	1.000	30,690	12,410
Profession (differences from intercept)	−0.166	1.753	−3.629	3.302	1.000	23,750	14,290
Training (differences from intercept)	1.051	1.658	−2.199	4.317	1.000	28,080	13,420
Education (differences from intercept)	1.314	1.376	−1.359	3.994	1.000	26,560	14,030
Using the internet (differences from intercept)	0.160	0.316	−0.458	0.775	1.000	34,500	13,360
Hosp.Specification (differences from intercept)	−1.435	1.926	−5.673	2.527	1.000	15,220	10,070
*Fixed Effects Estimates*							
Intercept	62.71	11.45	40.21	85.35	1.000	26,950	
DHC	−0.141	0.141	−0.416	0.134	1.000	25,080	
UI	0.807	0.077	0.656	0.958	1.000	30,610	
Profession	−0.166	1.765	−3.629	3.994	1.000	23,690	
Training	1.051	1.667	−2.199	4.317	1.000	27,850	
Education	1.314	1.367	−1.359	3.994	1.000	27,030	
Using Internet	0.160	0.315	−0.458	0.775	1.000	34,380	
Hosp.Spesification	−1.435	2.062	−5.673	2.577	1.000	14,490	
*Variance/Correlation Estimates*	Std.Deviation	Variance					
Hosp_ID Varian Estimates Intercept	2.501	6.254					
Residual Variance Estimates	20.38	415.2					

## 4 Discussion

This multi-hospital study provides multi-site evidence from Indonesia that perceived UI quality is the strongest proximal correlate of UX in routine hospital digital work. This study advances understanding of hospital digitalization by demonstrating that UI quality functions as a critical socio-technical interface layer linking healthcare infrastructure to frontline work practices. Previous studies emphasize that the electronic health record should align with individual goals as a person-centered approach, including individualizing information and orienting service plans (31). In this context, digital hospital systems are not merely information tools but components of complex infrastructures in which technological design, organizational context, and human work are tightly coupled.

This study reveals that the dominance of perceived UI quality as the most proximal correlate of UX should be interpreted as UI is not merely a cosmetic “front-end” but a socio-technical interface layer that mediates how hospital information infrastructures become usable or burdensome in the routines of healthcare workers. It is consistent with socio-technical Health Information Technology (HIT) models that treat the human-computer interface as an interdependent dimension alongside workflow/communication and organizational features (32, 33). Beyond individual experience, UI quality affects the performance and safety of healthcare infrastructure. Prior research demonstrates that poor EMR usability is associated with increased task load, workflow disruption, and higher risks of error, whereas usability improvements are linked to safer medication practices and reduced clinician strain (34, 35).

This study reveals that DHC has no detectable direct or indirect effects on UI/UX. It can be interpreted as compatible with established acceptance and Information System success traditions in which system quality and perceived ease-of-use are often the most immediate antecedents of user satisfaction and use, while individual attributes (e.g., self-efficacy, experience, and training) operate contingently and may not translate into better experience when interface constraints dominate interaction (36). Healthcare infrastructure research increasingly recognizes that system performance and care quality depend on how technologies are embedded in everyday workflows, not solely on their technical capabilities (31). The previous study emphasizes that digital interface competencies affect perceived user interface and user experience and further influence technology acceptance in health care (37).

The null DHC–UI/UX associations observed here are congruent with technology acceptance research. The Classic Technology Acceptance Model (TAM) and the Unified Theory of Acceptance and Use of Technology (UTAUT) synthesis show that perceived usefulness/performance and ease of use/effort expectancy, together with facilitating conditions (e.g., reliable devices, single sign-on, responsive support), explain the largest share of adoption and sustained use, so raising digital skills without fixing the interface yields diminishing returns for experienced quality (36, 38). Contemporary reviews of digital readiness in healthcare reinforce this pattern: while training helps, complex workflows, multiple logins, and misaligned design remain dominant barriers; the WHO likewise highlights digital health literacy as necessary but insufficient unless paired with needs-based design and organizational supports, a core PCC stance (16, 18, 39).

This study observed meaningful differences in DHC by profession, and, despite the small sample size among physicians, they tended to report higher UI/UX than larger groups (e.g., nurses, medical records officers). External evidence shows that usability and strain vary by specialty/profession and that poorer Electronic Health Record (EHR) usability is associated with higher odds of burnout, implicating workflow fit and task complexity rather than baseline digital skills (12, 13). Thus, profession-specific workflows and mental models plausibly account for the observed variability and are more tractable through co-design and iterative evaluation than through generic competency training alone.

This study reveals that item-level signals help prioritize actionable design work. Strengths clustered around clarity of language, consistency, and logical sequencing, while the lowest scores involved stability (errors), status visibility (progress indicators), on-screen help, and error localization/recovery. These gaps map directly to human-centered design principles in ISO 9241 210 (explicit understanding of users and contexts, iterative user-centered evaluation, and error prevention) and to well-established usability heuristics (e.g., visibility of system status; help, diagnosis, and recovery), indicating that usability engineering is a PCC strategy, not a cosmetic add-on. This standard is measured in terms of user experience (40, 41).

Literature shows that concrete EHR design features, including searchability, workflow fit, data entry support, user guidance, and automation, are causally linked to usability and medication safety, reinforcing that improving UI constitutes a safety intervention as well (42). From a workforce perspective, independent evidence links poor EHR usability to higher clinician stress and burnout, while incremental improvements in EHR usability are associated with lower odds of burnout. These relationships have been demonstrated across large national samples and varied specialties, aligning with PCC’s “care for the carers” imperative and supporting service sustainability (12, 13, 43). This is consistent with recent evidence that usability and safety-relevant EMR features vary across hospitals and vendors, with substantial between-institution variation in perceived usability and safety-related functionality (44).

UX research constitutes a methodical investigation of intended users and their specific needs, aimed at incorporating authentic contexts and insights into the design methodologies. The efficacy or ineffectiveness of a digital health innovation frequently hinges on how it is perceived by the end user (45). Accordingly, our findings suggest that hospital digitalization initiatives that prioritize infrastructure procurement without equal attention to UI quality risk under-deliver on both staff experience and the enabling conditions for PCC, whereas systematically strengthening UI quality may be among the most direct and scalable levers for making complex hospital infrastructures “work” for the people who use them, clinicians, and ultimately, patients.

In this study, UX was modestly higher in public hospitals, while UI and DHC were comparable across ownership. These results differ from previous research: participants rated person-centered care practices in private hospitals more highly, indicating a higher prevalence of these practices than in public hospitals (46). A previous study also proves that healthcare workers’ performance behavior is different depending on where they work (47). Hospital specification as PCC interpretation is that organizational “enabling environments”, governance, resources, and process coordination can shape experienced quality even when user skills are similar, a view embedded in IPCHS progress reports that highlight service responsiveness and co-production across settings (2). From a technology adoption perspective, facilitating conditions (e.g., infrastructure, single sign-on, support) are key determinants of acceptance and sustained use, and these often vary by organization/ownership; thus, systemic supports, not just user capability, likely explain the observed difference in UX (48, 49).

## 5 Conclusion

This multi-hospital study provides multi-site evidence from Indonesia that perceived UI quality is the most proximal and actionable determinant of UX in routine hospital digital work. Beyond confirming an empirical association, the study’s main theoretical contribution is to conceptualize UI as a socio-technical interface layer that links hospital information infrastructure to frontline work practices: the interface is where infrastructure is “felt” as either usable support or operational burden.

The prespecified mediation approach, combined with models that account for hospital-level clustering, yields the clear insight that DHC shows no detectable direct or indirect effect on UI/UX in this setting, whereas UX variation is predominantly at the individual level. Together, these results indicate that improving digital skills alone is unlikely to yield meaningful experiential gains if interface constraints remain; instead, UI quality constitutes the primary leverage point for enhancing staff experience, workflow reliability, and safety-sensitive performance in digital hospital systems. These findings carry important implications for both digital health practice and hospital digitalization policy. The proposed framework offers a pragmatic pathway from measurement to intervention by translating item-level UX insights into targeted design priorities, such as system stability and error handling, visibility of system status, contextual assistance, and recovery mechanisms. These priorities can be systematically integrated into procurement processes, acceptance testing, and ongoing post-implementation evaluation.

Future research should expand the framework through simulation-based human factors approaches to examine causal mechanisms linking specific UI improvements to outcomes such as workload, task efficiency, interruptions, and error recovery under realistic clinical conditions. Such investigations may employ workflow-sensitive simulation methods, including discrete-event, agent-based, or hybrid models, grounded in socio-technical representations of healthcare work systems and complemented by *in-situ* evaluations during infrastructure transitions. Finally, pragmatic implementation studies are needed to explore profession-specific workflows, governance structures, and enabling conditions across diverse hospital settings, as well as downstream service outcomes, in order to identify which UI-focused interventions can be scaled effectively and sustained within real-world healthcare digitalization efforts.

## 6 Limitations of the study

This study is cross-sectional; therefore, causal inference is not supported, and the mediation should be interpreted as a statistical partitioning of associations rather than a temporal mechanism. All measures (DHC, UI, UX) were self-reported at one time point, which increases susceptibility to common method variance, social desirability, and ceiling effects. In addition, scale alignment remains a concern: pragmatic UI and UX items may partially overlap conceptually (e.g., perceived ease/clarity/satisfaction elements), potentially inflating the apparent proximity of UI to UX. While instruments were applied consistently, further evidence of instrument validity in this setting is needed, especially discriminant validity between UI and UX and measurement invariance across professions and hospital types, because item interpretation may differ by role and context, and DHC may reflect general digital confidence rather than task- and system-specific competency.

Statistical and design features also restrict interpretation. The prespecified models assume correct functional form and omit potentially important confounders (e.g., system vendor/version/module, implementation maturity, workload intensity, shift patterns, and local support arrangements), which may bias estimates or mask true effects (particularly for DHC). Although clustering was examined, the nested structure (individuals within hospitals) and uneven cluster sizes can affect the precision of cluster-adjusted estimates and the stability of ICC values, especially when the number of hospitals is limited. Finally, the sampling approach limits generalizability and subgroup inference: professional composition was imbalanced (few physicians, many nurses), reducing power for profession-stratified comparisons and potentially introducing participation/availability bias. Future studies should use stratified recruitment across professions and hospitals, incorporate objective measures of interaction and performance alongside surveys, and explicitly model hospital- and system-level characteristics to strengthen inference about how UI quality, competency, and organizational enabling conditions jointly shape UX.

## Data Availability

The raw data supporting the conclusions of this article will be made available by the authors at https://sehariku.dinus.ac.id/, https://doi.org/10.60074/sehariku.v1i1.32 without undue reservation.
